# Comparative analysis of meat quality and flavor profiles between Bian chicken and Cobb broiler using FTIR and GC×GC-TOF MS technologies

**DOI:** 10.3389/fnut.2025.1665779

**Published:** 2025-09-22

**Authors:** Bochi Zhang, Rui Zhao, Kai Zhang, Wenjie Yu, Kaige li, Chunlei Yang, Liying Du, Kai Guo, Xianyi Song

**Affiliations:** ^1^College of Animal Science, Shanxi Agricultural University, Taiyuan, China; ^2^National Facility for Protein Science in Shanghai, Shanghai Advanced Research Institute, Chinese Academy of Sciences, Shanghai, China

**Keywords:** Bian chicken, breast muscle, FTIR, characteristic flavor compounds, breeds

## Abstract

This study systematically compared meat quality characteristics and flavor profiles between the local Shanxi breed Bian chicken (BIAN) and the commercial Cobb broiler (CB) using a comprehensive analytical approach. In terms of physical properties, Bian chicken exhibited significantly lower pH values (5.64 vs. 5.95; *p* = 0.026) and shear force (2.11 vs. 2.86 kg; *p* = 0.016), as well as a higher yellowness value b^*^ (7.37 vs. 5.66; *p* = 0.029), indicating better tenderness and meat color characteristics. Fourier transform infrared (FTIR) spectroscopy revealed fundamental differences in biomolecular composition and protein secondary structure between the two breeds. Specifically, Bian chicken showed higher α-helix content (37.54% vs. 33.50%), whereas Cobb had higher β-sheet content (35.45% vs. 32.39%) and greater total lipid (18.39% vs. 15.36%). These structural differences corresponded with the observed variations in meat quality traits. Comprehensive two-dimensional GC–TOF MS analysis showed that Bian chicken contained 2,150 volatile flavor compounds (including all detected isomeric forms), of which 1,068 were unique to Bian. The aldehyde content in Bian (15.13%) was notably higher than in Cobb (10.52%), and these low-threshold aroma compounds are crucial for a rich, meaty aroma. Differential compounds such as (E)-2-nonenal, 2-pentylfuran, heptanal, and 1-octen-3-ol were identified by multivariate analysis as potential markers distinguishing the two breeds. Sensory evaluation indicated that Bian chicken has more pronounced flavor characteristics, consistent with its unique compound profile. This study reveals significant biological differences in meat quality and flavor profiles between Bian chicken and Cobb broiler, demonstrating that Bian chicken possesses distinctive quality attributes including superior tenderness, unique protein structural characteristics, and a richer flavor profile dominated by key aroma compounds. These findings provide crucial scientific evidence for the conservation of indigenous breed characteristics, quality standardization, and market differentiation of Bian chicken, supporting its sustainable development and commercial valorization.

## 1 Introduction

China boasts a rich diversity of local chicken breeds. As living standards rise, consumer demand for superior chicken meat quality intensifies, making the study of meat quality traits and genetic mechanisms in these breeds a focal research area (1, 2). Recently, efforts to conserve and optimally utilize these valuable genetic resources have led researchers to evaluate and compare the germplasm characteristics and meat performance of local breeds. Numerous studies highlight significant differences in meat quality indicators between Chinese local breeds and commercial broilers (3). For instance, a study comparing Qingyuan Partridge Chickens (QPC) with Cobb (CB) broilers revealed that QPC exhibited lower pH values (by 6.88%), shear force (by 18.54%), moisture loss (by 30.71%), and muscle fiber area (by 53.96%), while showing higher meat color a^*^ (by 63.65%) and b^*^ values (by 150.27%), along with increased intramuscular fat (by 282.01%) (4). Similarly, comparisons of local breeds like Wenchang Chickens (WCH) and Xianju Chickens with commercial broilers (AV) demonstrated that slow-growing local breeds had higher shear values, inosine monophosphate concentrations, and fat contents, but lower moisture loss (5).

The flavor and texture of meat are the primary determinants of chicken quality. The distinct flavor profile of Chinese indigenous poultry breeds is largely attributed to their rich composition of organic flavor compounds. A study employing GC-IMS technology identified 65 organic flavor compounds, including 18 aldehydes, 16 alcohols, 10 ketones, and 9 esters, in soft-boiled meat from Mahuang and Tuer chickens (6). Previous research has characterized the key aroma compounds (nonanal, octanal, and dimethyl tetrasulfide) and breed-specific flavor compounds (hexanal, 1-octen-3-ol, (E)-2-nonenal, etc.) in chicken using GC-O and odor activity value analysis (7, 8). Furthermore, The GC-IMS method was employed to differentiate organic flavor compounds among six anatomical regions of Lueyang black chicken, identifying essential flavor compounds unique to each region. This study offers valuable insights for assessing the meat quality and flavor profile of Lueyang black chicken (9). A recent study utilizing GC×GC-TOF-MS on the breast muscles of four Chinese chicken breeds (Ningdu Yellow, Baier Yellow, Kangle, and Shengze 901) identified significant differences in 27 flavor compounds among these genotypes (10).

China is rich in local chicken breed resources, with consumers increasingly valuing their superior meat quality and distinctive flavor characteristics. Bian chicken is an indigenous breed from Shanxi Province in northern China, historically reared in areas along the Great Wall (11, 12). This breed is renowned for its superior meat quality, characterized by tender texture, rich flavor, and high nutritional value. Bian chicken meat contains abundant protein and favorable levels of flavor-enhancing compounds (such as amino acids and nucleotides), which contribute to its delicious taste and aroma (13). Consumers have a strong preference for native chickens like Bian chicken due to their unique flavor and mouthfeel, considering them a delicacy compared to conventional broilers (14). In fact, Bian chicken meat commands premium market prices and enjoys high consumer acceptance owing to these sensory advantages. Compared with fast-growing commercial broilers, Bian chickens consistently demonstrate better meat quality attributes in terms of taste, tenderness, and overall palatability. Additionally, Bian chickens are hardy and well-adapted to free-range systems, thriving on coarse feed and in cold climates (15). These favorable characteristics have made Bian chicken a focus of food science and agricultural research, as researchers seek to capitalize on its exceptional flavor, nutrition, and consumer appeal.

Although numerous studies have explored the growth traits, reproductive performance, and genetic markers of Bian chickens, limited research has been conducted on their flavor characteristics and meat quality attributes to date. Cobb broiler was selected as the comparator because it represents the global commercial standard, with intensive selection for rapid growth providing maximum genetic and phenotypic contrast to indigenous Bian chickens. Fourier transform infrared spectroscopy (FTIR) rapidly fingerprints protein–lipid vibrations in chicken, enabling chemometric mapping of lipid-oxidation precursors that shape aroma; it is non-destructive, high-throughput, and needs minimal preparation, with reproducible, interpretable outputs (16). GC×GC-TOF MS offers two-dimensional separation with high peak capacity and fast detection, resolving coeluting volatiles and quantifying key odorants to characterize flavor profiles across muscles, treatments, and processing stages (17). Combined, FTIR screens changes while GC×GC-TOF MS elucidates volatile drivers, delivering comprehensive, robust flavoromics for chicken meat quality research. This study aims to systematically compare the biomolecular composition, protein secondary structure, and flavor compounds of Bian chicken and commercial Cobb broiler meat in order to comprehensively understand the unique meat quality and flavor profile of Bian chicken. These findings are significant for breed conservation and for meeting consumers' demand for high-quality traditional poultry products.

## 2 Materials and methods

### 2.1 Animal and sample information

Animal experiments adhered to the Guide for the Welfare and Use of Laboratory Animals (Ministry of Science and Technology of the People's Republic of China, 2006). To ensure consistent meat quality evaluation, only female animals were used. Bian chickens (an indigenous chicken breed) were sourced from Shanxi Nongkang Xintuo Technology Development Co., Ltd., and Cobb broilers (commercial Cobb 500 strain) from Shanxi Daxiang Agriculture and Animal Husbandry Technology Co., Ltd. Post-hatching, 80 healthy 1-day-old Bian chickens and 80 Cobb chickens were selected and raised under identical conditions at Shanxi Agricultural University's practice farm. Birds were divided into 10 replicate groups (10 cages per breed, 4 birds per cage). The chickens had unrestricted access to feed and water. They were housed individually in cages and managed according to guidelines (GB/T 5916−202, NY/T 1871−201). The rearing temperature was maintained at 35 °C during the first week and then gradually lowered by ~0.5 °C per day until stabilizing at 21–26 °C. Relative humidity was maintained at 65%−70% during days 1–7, 60%−65% during days 8–14, and 50%−60% from day 15 onwards. The diet for Cobb broilers contained 21.5% crude protein and 12.1 MJ/kg metabolic energy from 1 to 21 days of age, and 17.5% crude protein and 12.4 MJ/kg from 22 to 42 days. The study was conducted in accordance with the Chinese Guidelines for Animal Welfare, and approved by the Ethics Committee of Shanxi Agricultural University (protocol code SXAU-EAW-2024C.YC.007010312).

### 2.2 Sample collection

All chickens were slaughtered at their respective market ages. The Cobb chickens were fed for 42 days and the Bian chickens for 112 days. Feed was withheld for 16 h and water for 12 h prior to slaughter. Eight chickens per group (one per replicate) were slaughtered, and ~30 g of pectoralis muscle was collected from similar anatomical sites in each bird. The samples were allocated as follows: 10 g for immediate meat quality measurements (pH, color, shear force, and moisture loss), 3 g for GC × GC-TOF MS analysis, and 5 g for FTIR spectroscopy. The remaining tissue was stored as backup. Samples designated for GC × GC-TOF MS and FTIR analyses were placed in sterile tubes, rapidly frozen in liquid nitrogen, and stored at −80 °C until analysis.

### 2.3 Measurement of meat quality traits

Various measurements were conducted to evaluate meat quality, including pH value, meat color (a^*^, b^*^, L^*^), shear force, and moisture loss. All 10 samples were selected for the measurement of pH value, meat color, shear force, and moisture loss. Each sample was measured 3 times, and the average value was taken. The determination of each meat quality index followed the guidelines outlined in the “Work Manual for the Third National Census of Livestock and Poultry Genetic Resources (First Edition)” (18). Meat color (L^*^, a^*^, b^*^) was measured using a Chroma Meter CR-410 (Konica Minolta, Japan) at 24 h postmortem at room temperature. Three locations on each breast muscle sample were measured in triplicate (nine readings total per sample), and the average values were calculated. The Shear force was measured with a meat tenderometer (C-LM4, Tenovo, Beijing, China) at a speed of 200 mm/min. Take a piece of fresh pectoral muscle and cut it into strips, 1.0 cm wide and 0.5 cm thick, to ensure removal of tendons, fat and membranes, cross section perpendicular to the direction of muscle fibers, each cut three times, record the average value. pH detection was performed using a portable pH meter (Testo 205, Testo AG, Germany) equipped with a penetration electrode. The electrode was inserted ~1 cm deep into three different locations of the pectoralis muscle, avoiding fat and connective tissue. The pH of the pectoral muscle was recorded three times at each location after slaughter and the average of these readings was calculated. Moisture loss is measured by weighing about 1.5 g pectoral muscle, removing tendons, fat, and membranes, and slicing it. The sections were placed between two stacks of 18-layer filter paper and then sandwiched between two hard plastic plates. Apply a pressure of 35 kg for 5 min, then remove and immediately weigh the sample.

### 2.4 Fourier transform infrared spectroscopy

Biomolecular concentration changes in meat samples were measured using attenuated total reflection (ATR)–FTIR spectroscopy. This employed a single-reflection ATR module on a Bruker Tensor 27 FTIR spectrometer (Bruker Optics Ltd., Ettlingen, Germany) covering a spectral range of 4,000–500 cm^−1^ with a resolution of 4 cm^−1^, collecting 64 scans per sample. Spectra acquisition and instrument control were managed using OPUS software (version 7.2, Bruker Optics Ltd, Germany). The infrared spectra were analyzed to extract information on components of interest, including their relative quantification and secondary structure.

Ten samples each of BIAN and CB were selected to study changes in biomolecular content. Each group originally consisted of 150 spectra (15 per sample), which were transformed into second-order derivatives using 13 smoothing points. These were then averaged to reduce the data to 3 spectra per sample. Data processing involved peak marking, baseline correction, and normalization with Omnic 8. The processed spectra were fitted using the GaussAmp function in Peakfit 4.12, and spectral mapping was conducted with Origin 2017.

Spectral data underwent second-order derivative preprocessing to resolve adjacent peaks and enhance features. We concentrated on spectral regions of 3,000 to 2,800 cm^−1^ and 1,800 to 900 cm^−1^, applying second-order derivatives and normalization to calculate integrated areas for each functional group. OPUS software (version 7.2, Bruker Optics GmbH, Germany) was used to integrate processed spectra from 10 BIAN and 10 CB samples across various regions to ascertain biomolecular proportions. A nonlinear least-squares curve fitting technique, employing Gaussian and Lorentzian functions, analyzed overlapping peak areas of the amide I band in FTIR. Depending on sample types, fitting parameters such as β-sheet, α-helix, β-turn, and antiparallel structures, along with peak positions and band shapes, were derived from the FTIR spectra.

### 2.5 Two-dimensional gas chromatography-time of flight mass spectrometry (GC×GC-TOF MS)

An appropriate amount of chicken breast freeze-dried sample was taken into a 20 ml headspace injection vial, and add 10 μl of internal standard solution for quantitative calibration. Samples were incubated at 80 °C for 10 min to allow sufficient release of volatile compounds into the headspace. SPME extraction heads are aged at 270 °C for 10 min prior to use to remove possible contaminants. The aged SPME extraction head is inserted into the headspace of the sample bottle and adsorbed at 80 °C for 25 min to ensure adequate concentration of volatile compounds. After adsorption, transfer the SPME extraction head to the gas chromatography injection port and thermally desorb at 250 °C for 5 min to completely release the adsorbed compounds into the chromatographic system. After each sample injection, the SPME extraction head is aged at 270 °C for 10 min in preparation for the next analysis. To calculate the retention index, an additional 10 μl of n-alkane standard was placed in a 20-ml headspace injection vial and incubated, extracted, and injected for analysis under the same conditions. The meat samples were analyzed using a LECO Pegasus BT 4D system, which included an Agilent 8890A gas chromatograph, a dual-stage jet modulator, and a split/splitless injection module for GC-TOF MS analysis. A high-resolution TOF mass detector was utilized for mass spectrometry. Two columns were employed for separation: a DB-Heavy Wax column (30 m × 250 μm × 0.5 μm) and an Rxi-5Sil MS column (2 m × 150 μm × 0.15 μm). Helium gas of high purity was the carrier at a constant flow rate of 1.0 mL/min. The temperature program for the DB-Heavy Wax column started at 50 °C, held for 2 min, then increased to 230 °C at a rate of 5 °C /min, and held for 5 min. The Rxi-5Sil MS column was maintained 5 °C higher than the DB-Heavy Wax column. The modulator temperature was consistently set 15 °C above the Rxi-5Sil MS column, with a modulation period of 6.0 s. The injector temperature was kept at 250 °C. The LECO Pegasus BT 4D mass detector had a mass transfer line temperature of 250 °C and an ion source temperature. Data processing was performed using ChromaTOF software (version 5.0, LECO Corporation). Raw data preprocessing included baseline correction using a median filter (10-point window) and peak deconvolution with an automated peak find algorithm (S/N threshold of 100:1). Peak areas were quantified using unique mass fragments and normalized to the total ion chromatogram. Compound identification was achieved through spectral matching against the NIST 2020 Mass Spectral Library. Only compounds detected in ≥80% of biological replicates were retained for statistical analysis.

### 2.6 Statistical analysis

Independent samples *t*-tests were used to compare meat quality parameters (pH, color, shear force, moisture loss) and FTIR spectral data between BIAN and CB groups, with Bonferroni correction applied for multiple comparisons in FTIR analysis (adjusted α = 0.01). For GC×GC-TOF MS data, multivariate analyses (PCA, PLS-DA, OPLS-DA) were performed using SIMCA-P (version 14.1, Umetrics, Sweden) after log transformation and Pareto scaling, with differential compounds identified using fold change criteria (FC > 2 or < 0.5) and FDR-corrected *t*-tests (*q* < 0.05). Statistical significance was set at p^*^ < 0.05, p^**^ < 0.01, and p^***^ < 0.001, with all analyses performed using GraphPad Prism 7.0 and R software (version 4.2.1), and data presented as mean ± SEM.

## 3 Results and discussion

### 3.1 Analysis of differences in meat quality traits between BIAN and CB

To investigate the meat quality differences between BIAN and CB chicken breasts, we assessed key meat characteristics. Physical properties were measured (Table 1), revealing that the pH of CB was significantly higher than that of BIAN (*p* < 0.05). Although no significant difference was observed in the L^*^ value, the b^*^ value of BIAN was significantly higher than that of CB (*p* < 0.05), suggesting a more reddish hue in BIAN meat compared to the paler CB. Additionally, BIAN exhibited a significantly lower shear force than CB (*p* < 0.05). Overall, BIAN demonstrated a lower pH, reduced shear force, and a higher b^*^ value in comparison to CB, indicating notable differences in meat quality.

**Table 1:** Summary statistics of meat quality traits of BIAN and CB.

**Traits**	**BIAN**	**CB**	***p*-value**
pH_24h_	5.64 ± 0.06	5.95 ± 0.13	0.026^*^
**Color**_24h_			
${\text{L}}_{24\text{h}}^{*}$	52.42 ±0.97	53.88 ± 2.19	0.351
${\text{a}}_{24\text{h}}^{*}$	1.83 ± 0.32	1.72 ± 0.19	0.636
${\text{b}}_{24\text{h}}^{*}$	7.37 ± 0.66	5.66 ± 0.60	0.029^*^
Moisture loss, %	26.48 ± 2.39	28.73 ± 1.64	0.622
Shear force, kgf	2.11 ± 0.18	2.86 ± 0.27	0.016^*^

BIAN, Bian chicken; CB, Cobb broiler chicken. ^*^p < 0.05.

This study revealed significant differences in meat quality characteristics and flavor profiles between BIAN chickens and Cobb broilers (CB), indicating that breed fundamentally influences the physicochemical properties and flavor compounds composition. pH is a crucial metric for assessing meat quality, with chicken breast meat typically ranging from 5.8 to 5.9. Elevated pH levels can impart undesirable flavors when the meat is boiled. The higher pH in CB chickens (5.95 vs. 5.64, *p* < 0.05) aligns with previous observations in fast-growing broilers, where rapid muscle growth and altered glycolytic metabolism hinder postmortem pH decline (19). This pH variation may explain the observed color changes. BIAN chickens show significantly higher b^*^ values (7.37 vs. 5.66, *p* < 0.05), consistent with studies on local breeds that exhibit increased yellowness due to elevated myoglobin content and oxidative muscle fiber composition (20). The notably lower shear force in BIAN chickens (2.11 vs. 2.86 kg, *p* < 0.05) indicates that the distinct muscle fiber and collagen traits of local breeds may mitigate age-related changes (21). BIAN chicken exhibits distinctive quality attributes that set it apart from both commercial broilers and other native breeds, particularly its exceptionally low shear force (2.11 kg) despite extended rearing (112 days) and significantly higher b^*^ value (7.37), which exceeds not only Cobb broilers (5.66) but also surpasses values reported for other premium native breeds like Guangyuan Gray chicken (22). These unique physical properties position BIAN chicken as a distinctive premium product with strong market potential for quality-conscious consumers.

### 3.2 Determination of the proportions of biomolecules and secondary structure proteins in BIAN and CB chicken breasts by FTIR characterization

**Figure 1:**
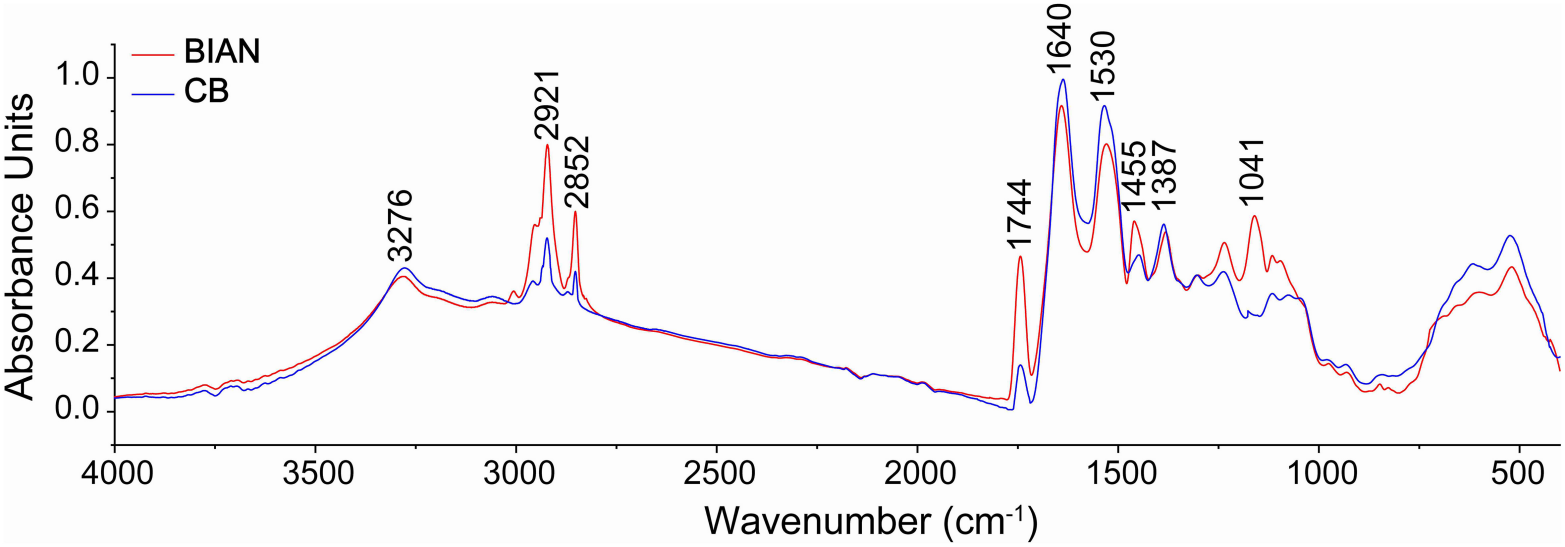
Comparison of the average original spectra of BIAN and CB chicken breast samples. Infrared spectra were detected in the spectral region of 4,000–500 cm^−1^, resolution 4 cm^−1^, based on 64 scans.

**Figure 2:**
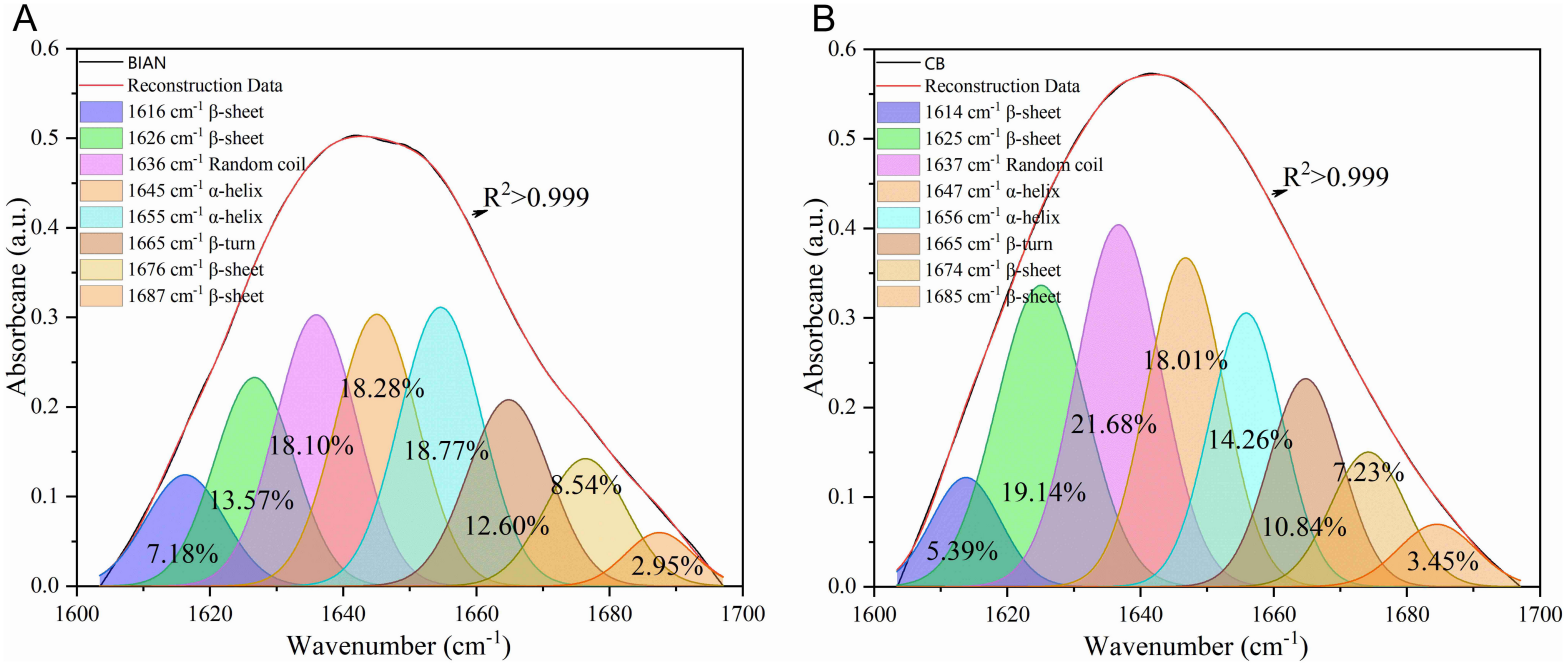
Deconvolution results of amide I bands in six chicken breast samples of BIAN and CB chicken. **(A)** BIAN samples; **(B)** CB samples.

Chicken samples primarily consist of proteins, fats, and trace amounts of carbohydrates and inorganic salts. Their infrared spectra predominantly reflect the vibrational modes of functional groups within these components. The spectral consistency across samples suggests that the structural composition of chicken is stable, with proteins as the main component, complemented by fats and polysaccharides. As shown in Figure 1, the two breeds share conserved band positions across 4,000–500 cm^−1^, but their relative intensities differ: CB displays stronger lipid-related C–H stretching at ~2,921 and 2,852 cm^−1^, whereas BIAN shows higher amide I (~1,640 cm^−1^) and amide II (~1,530 cm^−1^) bands; the 1,250–900 cm carbohydrate region is comparable between groups. Key absorption peaks are observed at 3,276 cm^−1^ (amide A band, O–H and N–H stretching), 2,921 and 2,852 cm^−1^ (lipid, alkyl C–H stretching), 1,640 cm^−1^ (amide I band, C=O stretching), 1,530 cm^−1^ (amide II band, N–H bending and C–N stretching), and 1,041 cm^−1^ (polysaccharide C-O stretching). The amide I band (1,700–1,600 cm^−1^), a key vibrational signature of the protein backbone, reflects secondary structural elements. Figure 2 demonstrates that deconvolution of this band reveals notable differences in secondary structure between the groups. As detailed in Table 2, the α-helix content is significantly higher in BIAN samples (37.54 ± 0.34%) compared to CB samples (33.50 ± 1.46%, *p* = 0.019). Conversely, the β-sheet content is greater in CB samples (35.45 ± 2.10%) than in BIAN samples (32.39 ± 0.28%). No significant differences are observed in β-turn and random coil structures: BIAN samples show 11.80 ± 0.84% and 18.27 ± 0.38%, respectively, while CB samples have 11.21 ± 0.64% and 19.83 ± 1.35%.

**Table 2:** The proportion of secondary structure proteins in BIAN and CB chicken breast samples (percentage of curve fitting ± standard error).

**Secondary protein structure (wavenumber)**	**% Curve fitting** ±**standard error**		***p*-value**
	**BIAN**	**CB**	
Alpha helix	37.54 ± 0.42	33.50 ± 1.78	0.019^*^
Beta sheet	32.39 ± 0.35	35.45 ± 2.57	0.111
Beta turn	11.80 ± 1.03	11.21 ± 0.79	0.479
Random coil	18.27 ± 0.47	19.83 ± 1.65	0.190

BIAN, Bian chicken; CB, Cobb broiler chicken. ^*^p < 0.05.

Analysis of biomolecular composition using specific spectral regions identified notable differences in lipid and protein contents between the two chicken breeds. Table 3 shows that the CB samples had a significantly higher total lipid content (18.39 ± 0.27%) compared to the BIAN samples (15.36 ± 0.25%, *p* < 0.001), with pronounced differences in ester lipid content (CB: 10.41 ± 0.28% vs. BIAN: 6.64 ± 0.21%, *p* < 0.001). Conversely, BIAN samples exhibited higher protein peaks, with an amide I content of 22.63 ± 1.08% vs. 19.78 ± 0.53% for CB (*p* = 0.02), and an amide II content of 20.31 ± 0.60% vs. 18.77 ± 0.48% for CB (*p* = 0.03). Carbohydrate content in the 1,250–900 cm^−1^ region showed no significant difference between groups (BIAN: 16.36 ± 1.71%, CB: 14.69 ± 0.74%, *p* = 0.20). These spectral differences were consistent across repeated measurements, underscoring the reproducibility of FTIR spectroscopy in differentiating chicken types by molecular composition.

**Table 3:** Ratio of biomolecules determined by Fourier transform infrared in chicken breast samples determined from BIAN and CB (percentage of integral area ± standard errors).

**Biomolecule (wavenumber)**	**% Integral area**		***p*-value**
	**BIAN**	**CB**	
Lipid (3,000–2,800 cm^−1^)	15.36 ± 0.25	18.39 ± 0.27	0.00^**^
Ester lipid (1,740 cm^−1^)	6.64 ± 0.21	10.41 ± 0.28	0.00^**^
Amide I (1,700–1,600 cm^−1^)	22.63 ± 1.08	19.78 ± 0.53	0.02^*^
Amide II (1,600–1,500 cm^−1^)	20.31 ± 0.60	18.77 ± 0.48	0.03^*^
Carbohydrate (1,250–900 cm^−1^)	16.36 ± 1.71	14.69 ± 0.74	0.20

BIAN, Bian chicken; CB, Cobb broiler chicken. ^**^p < 0.001.^*^p < 0.05.

Differences in protein secondary structures between BIAN and CB chicken breeds significantly impact meat quality, aligning with studies linking α-helix structures to enhanced tenderness and juiciness (23). BIAN chickens exhibited a higher α-helix content (37.54%) compared to CB chickens (33.50%), suggesting softer meat due to the α-helix's flexible and elastic conformation, which enhances texture (24). This observation corroborates synchrotron-FTIR research showing that increased α-helix content correlates with improved tenderness in chicken meat (25). Additionally, CB chickens had a notably higher total lipid content than BIAN (18.39% vs. 15.36%, *p* < 0.001), and a greater ester lipid fraction (10.41% vs. 6.64%, *p* < 0.001), as shown by the FTIR integral areas in Table 3. These lipid differences helped explain breed-dependent patterns of lipid metabolism and deposition (26). The elevated protein levels in CB chickens, indicated by the intensified amide I and II bands, underscore the focus of commercial breeding on lean meat yield and rapid muscle growth. However, this does not necessarily equate to superior eating quality, as protein levels do not always correlate with tenderness or juiciness (27). The comparable carbohydrate content between the breeds suggests similar glycogen metabolism and post-mortem glycolysis, implying that meat quality differences primarily arise from variations in proteins and lipids rather than carbohydrates. The biomolecular differences revealed by FTIR analysis provide critical insights into the superior meat quality of Bian chicken. The higher α-helix content (37.54%) in Bian chicken directly correlates with its improved tenderness (lower shear force of 2.11 kg), while the higher protein content (amide I: 22.63% and amide II: 20.31%) contributes to its distinctive texture characteristics. Despite having lower total lipid content (15.36% vs. 18.39% in Cobb), Bian chicken's unique protein structure compensates by enhancing meat tenderness and maintaining juiciness. These structural characteristics not only explain why local consumers prefer Bian chicken but also provide objective quality markers for breed authentication and preservation programs. Such molecular-level understanding is essential for maintaining the genetic integrity of this indigenous breed and meeting consumer demand for high-quality traditional poultry products (28).

### 3.3 Analysis of the differences in flavor compounds between BIAN chicken and Cobb broilers

**Figure 3:**
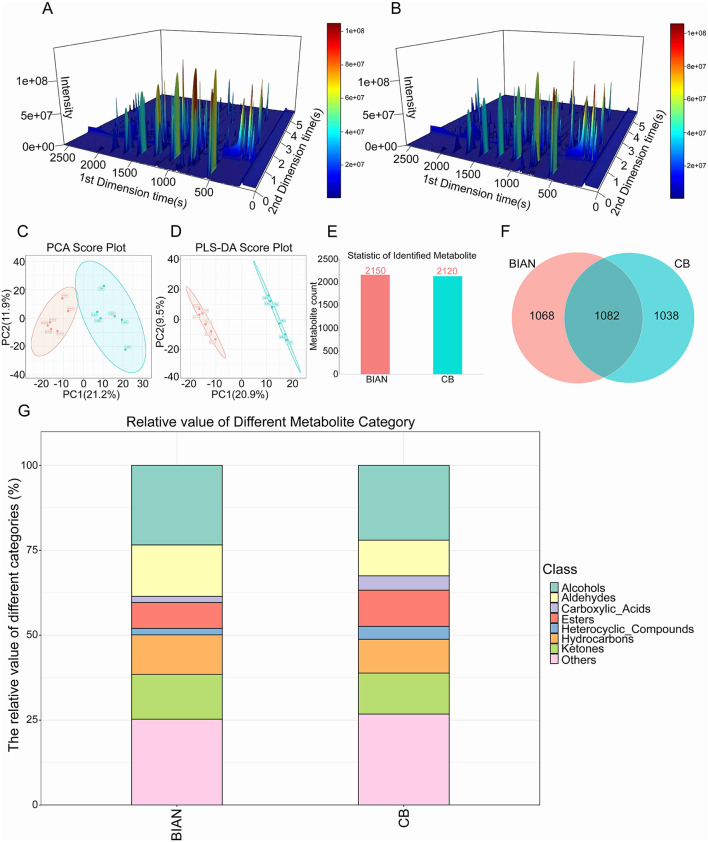
Flavor compound differences between BIAN chicken and Cobb broilers: **(A,B)** 3D total ion chromatograms of BIAN and Cobb chickens; **(C,D)** PCA and PLS-DA plots of 12 samples; **(E)** Number of identified components; **(F)** Venn diagram of component identification; **(G)** Relative content of flavor compound types in breast meat.

GC×GC-TOF MS total-ion chromatograms (Figures 3A, B) showed abundant peaks in both breeds, with Bian chicken exhibiting denser and higher-intensity peak clusters indicative of breed-specific chemical profiles. This chromatographic pattern is consistent with the higher aldehyde fraction in BIAN (15.13% vs. 10.52%) and the stronger fruitiness observed in sensory evaluation.

Multivariate statistical analysis confirmed this distinction. The PCA score plot revealed complete separation of the two breeds along the first principal component (PC1). As depicted in Figures 3C, D, Bian chicken samples clustered within the pink ellipse in P1′s negative region, while Cobb broiler samples were within the blue ellipse in the positive region. Each group's samples were tightly clustered, with a clear boundary, indicating systematic differences in components and strong biological reproducibility. PLS-DA improved discrimination by incorporating breed information, resulting in a more polarized distribution: Bian chickens clustered toward the lower-left, Cobb broilers toward the upper-right, significantly increasing the distance between groups. The OPLS-DA score plot further enhanced group separation, displaying a more pronounced bipolar distribution on the horizontal axis.

This study identified notable differences in flavor profiles between Bian and Cobb chickens. Through comprehensive GC×GC-TOF MS analysis, we found that genetic background significantly influences meat flavor characteristics. Specifically, 2,150 flavor compounds were detected in Bian chickens, compared to 2,120 in Cobb broilers, with 1,068 and 1,038 compounds unique to each breed, respectively. This suggests variations in metabolic pathways affecting flavor development. These findings align with recent studies highlighting breed-specific differences in poultry meat quality (29, 30). Multivariate statistical analyses using PCA and PLS-DA confirmed distinct and reproducible flavor differences between the breeds. This approach parallels the multi-omics analysis of Qingyuan Partridge chickens and Cobb broilers, which also demonstrated clear breed separation (31).

After annotating and purifying, the flavor compounds in Bian chickens and Cobb broilers were identified. Figures 3E, F illustrates 2,150 flavor compounds in Bian chickens and 2,120 in Cobb broilers. Bian chickens displayed a higher count of flavor compounds within the detectable range. Through statistics, it was found that there are a total of 1,082 flavor compounds between these two varieties. Additionally, Bian chickens possessed 1,068 exclusive flavor compounds, while Cobb broilers had 1,038 unique compounds. Consequently, Bian chickens showcased a more diverse array of flavor compounds compared to Cobb broilers.

Using the database, we identified flavor compounds in the breast meat of Bian chickens and Cobb broilers, revealing significant differences in aroma compound types. As illustrated in Figure 3G, Bian chickens had a higher alcohol content (23.4%) compared to Cobb broilers (22.0%), contributing fresh, floral, and fruity odors. Notably, aldehydes in Bian chickens were 15.13%, markedly higher than the 10.52% in Cobb broilers, significantly enhancing meaty, fatty, and nutty aromas due to their low threshold. Hydrocarbons (11.64% vs. 9.95%) and ketones (13.18% vs. 12.09%) were also more prevalent in Bian chickens, with ketones imparting creamy and floral scents. Cobb broilers exhibit higher levels of carboxylic acids (4.21% vs. 1.82%), esters (10.69% vs. 7.60%), heterocyclic compounds (3.77% vs. 1.94%), and other flavor compounds (26.78% vs. 25.27%). Carboxylic acids are typically associated with sour and pungent odors, whereas esters impart fruity and sweet aromas. Heterocyclic compounds, including pyrazines and thiazoles, are key Maillard reaction products that generate roasted meat and nutty aromas. These variations suggest that Bian chickens may possess a more complex fruity and roasted flavor profile. The distinct compositions of components in these chicken types underpin their unique flavor characteristics. Aldehydes constitute 14%−26% of the flavor compounds in chicken, with variations depending on the breed. They are pivotal in differentiating flavor profiles among breeds due to their low odor thresholds, contributing to the meaty, fatty, and nutty flavors in poultry (10). An analysis of 972 Chinese native chickens revealed that native breeds have the highest aldehyde content, which is inversely related to other compound categories, underscoring aldehydes as primary flavor determinants (32).

### 3.4 Screening of differential compounds between Bian chickens and Cobb broilers

**Figure 4:**
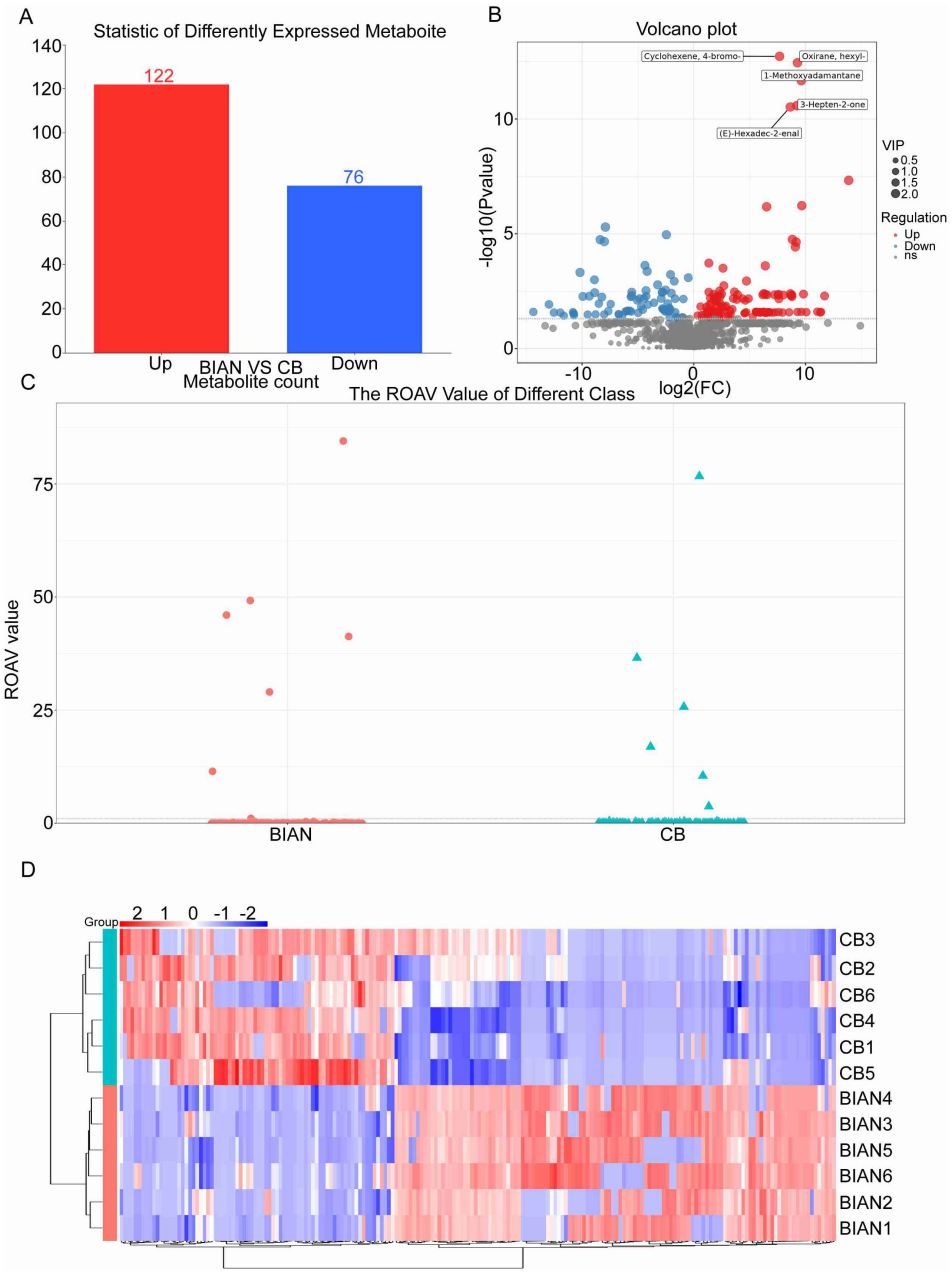
Flavor compound screening in BIAN and CB chickens: **(A)** number of differential flavor compounds; **(B)** volcano plot; **(C)** ROAV odor activity value scatter plot; **(D)** differential metabolite heatmap.

A comprehensive analysis of differential compounds between BIAN and CB chicken breeds uncovered significant flavor differences using multiple analytical methods. Numerous differential flavor compounds were identified. As depicted in Figure 4, compared to Cobb broilers, 122 flavor compounds were up-regulated, while 76 were down-regulated in Bian chickens. The volcano plot illustrates the statistical significance and magnitude of changes in flavor compounds between BIAN and CB, with the horizontal axis representing log2 fold change and the vertical axis showing -log10(*p*-value). Red dots indicate significantly up-regulated compounds in BIAN, blue dots represent significantly down-regulated compounds, and gray dots denote no significant differences. The heatmap reveals a breed-specific expression pattern of differential flavor compounds, and the dendrogram confirms excellent reproducibility within breeds and fundamental metabolic differences between them.

Table 4 highlights the distinctive flavor compounds in Bian chicken meat, including (E)-2-Nonenal, 2-pentyl-Furan, Heptanal, (E)-2-Octenal, 1-Octen-3-ol, and 2-methyl-Butanal. The high concentrations of these compounds likely contribute to the unique flavor profile of Bian chickens. Based on differential metabolite analysis and relative odor activity, (E)-2-Nonenal, 2-pentyl-Furan, Heptanal, and 1-Octen-3-ol are suggested as key markers distinguishing Bian chickens from Cobb broilers. (E)-2-Nonenal imparts a tallowy taste with an oily odor crucial to chicken breast flavor; 2-pentyl-Furan offers floral, fruity, and earthy notes; Heptanal contributes potato and roasted flavors, serving as a primary flavor compound for meat flavor; 1-Octen-3-ol, with its mushroom-like aroma, is a common flavor contributor in various meats, including poultry. Notably, (E)-2-nonenal, 2-pentylfuran, and heptanal have been identified as key flavor compounds, potentially distinguishing Bian from Cobb chickens. These compounds, products of lipid oxidation, significantly influence meat flavor. (E)-2-nonenal is especially crucial for breed differentiation. A study comparing Qingyuan partridge chicken to commercial broilers showed significantly higher odor activity values (OAV) of (E)-2-nonenal in native chicken soup than in broiler chicken soup (OAVs of 120 vs. 39), highlighting its importance for chicken soup aroma (33). Heptanal consistently emerges as a key discriminatory compound in comparative studies of various chicken breeds. Research using gas chromatography-ion mobility spectrometry (GC-IMS) identifies heptanal as a primary flavor compound in Chinese chicken breeds from different regions (34). Its concentration varies with breed genetics and slaughter age (35). 2-Pentylfuran, formed through complex thermal reactions during cooking, imparts unique fruity, floral, and caramel-like flavors to chicken. Recent studies have elucidated its formation pathway and quantified its sensory significance (36). Breed-specific accumulation of these compounds in Bian chickens suggests differences in enzyme expression related to lipid oxidation and Maillard reaction pathways, a view supported by recent transcriptomic studies on local chicken breeds (37).

**Table 4:** Comparison of ROAV of flavor compounds in BIAN and CB chicken breast.

**Item**	**Class**	**CAS**	**BIAN**	**CB**
(E)-2-Nonenal	Aldehydes	18829-56-6	84.50	36.56
2,3-Butanedione	Ketones	431-03-8	49.22	76.70
2-pentyl-Furan	Organoheterocyclic compounds	3777-69-3	46.03	16.90
Heptanal	Aldehydes	111-71-7	41.28	25.72
(E)-2-Octenal	Aldehydes	2548-87-0	29.02	10.48
1-Octen-3-one	Ketones	4312-99-6	11.47	3.71
2-methyl-Butanal	Aldehydes	96-17-3	1.07	0.42

BIAN, Bian chicken; CB, Cobb broiler chicken.

### 3.5 Sensory flavor analysis of Bian chickens and Cobb broilers

**Figure 5:**
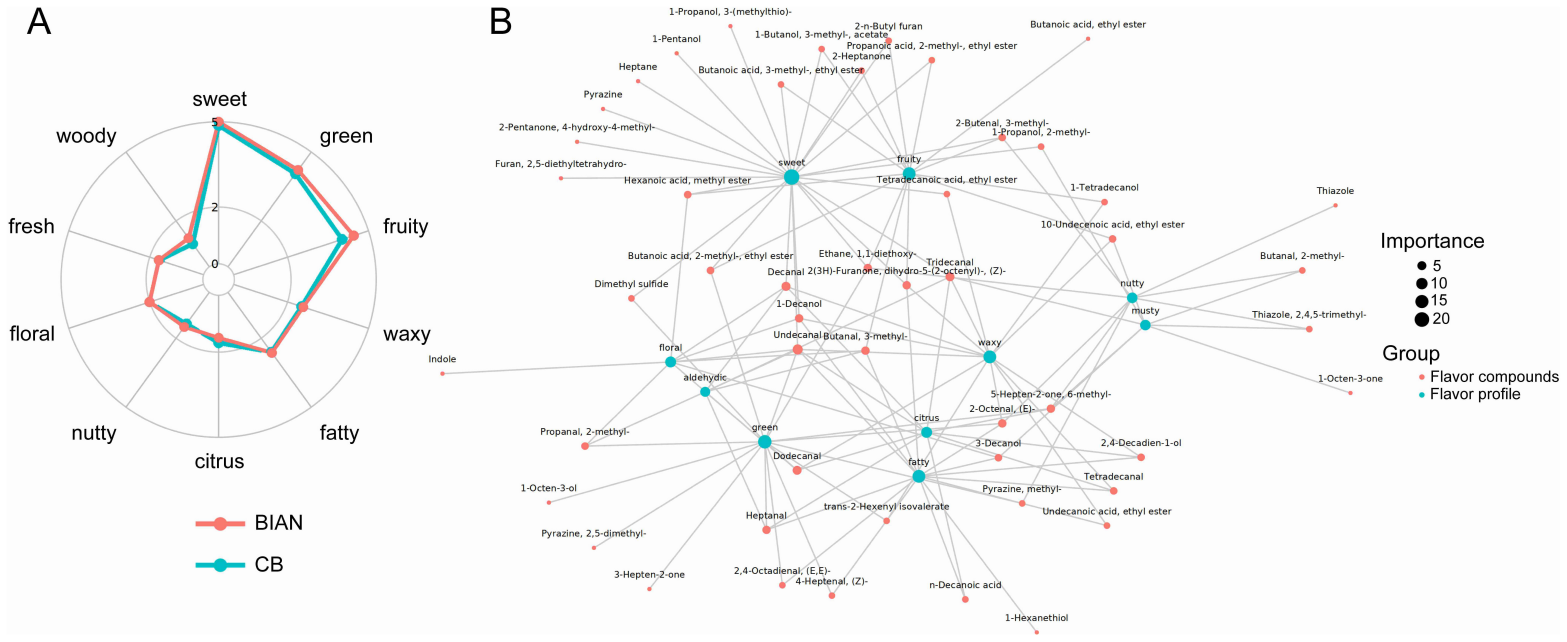
**(A)** Radar chart for sensory flavor characteristic analysis. **(B)** Correlation network diagram between sensory and flavor compounds.

The radar chart of sensory flavor analysis reveals the similarities and significant differences between BIAN and CB in ten flavor attributes. As can be seen from Figure 5A, the two varieties displayed distinct differences in their flavor profiles. Sweetness, greenness, and fruitiness were the most intense attributes, while woody, fresh, and citrus notes were minimal. A notable disparity was observed in fruitiness, with BIAN exhibiting significantly higher intensity than CB, suggesting that BIAN may have more pronounced fruity flavor compounds. Both varieties showed moderate and nearly identical levels of floral, nutty, and waxy attributes, indicating similar complexity in these dimensions. This similarity suggests that the fundamental flavor characteristics are retained across the varieties, and BIAN's enhanced fruitiness aligns with metabolomic findings of variety-specific compounds. Using igraph network analysis, a network diagram was constructed to illustrate the relationship between flavor compounds and sensory characteristics. Radar chart analysis indicates that Bian chickens, in contrast to Cobb chickens, exhibit more pronounced fruity, nutty, and woody aromas, while maintaining comparable levels of sweetness, floral notes, and fatty characteristics. This intricate sensory profile is consistent with consumer preference studies in Asia. Research suggests that Korean consumers perceive native chicken breeds as offering a more flavorful taste, juicier texture, and desirable chewiness, with breed information serving as a critical sensory cue influencing purchasing decisions (38).

In Figure 5B, red circles denote flavor compounds, with larger circles indicating more associated sensory characteristics. Green circles represent sensory attributes, with their size reflecting the diversity of connected flavor compounds. In Bian chickens, (E)-2-Nonenal was linked to the fatty flavor; 2-pentyl-Furan was associated with coffee, onion, and sulfury notes; Heptanal was connected to fatty, herbal, aldehydic, green, and fresh flavors; 1-Octen-3-ol was linked to chicken, green, oily, mushroom, and fishy flavors. The detection of two types of chicken using GC×GC-TOF MS indicates that Bian chickens have their unique flavors, which is an important reason why Chinese consumers are more receptive to them. Similarly, studies employing gas chromatography-mass spectrometry have demonstrated that traditional processing methods for Chinese poultry products significantly enhance these favorable flavor compounds (39). Network analysis linking specific compounds to sensory attributes provides critical insights for quality control. These findings hold substantial significance for the preservation of local breed characteristics. Globally, local chicken breeds possess unique quality attributes that are highly valued by local consumers (40). This study integrates multiple research methodologies, including FTIR for protein structure analysis and GC×GC-TOF MS for flavor compounds analysis, offering a comprehensive understanding of meat quality.

## 4 Conclusions

This study systematically characterized the meat quality and flavor differences between Bian chicken and Cobb broiler through integrated analytical approaches. Bian chicken exhibited superior meat quality attributes including lower pH (5.64 vs. 5.95) and shear force (2.11 vs. 2.86 kg), indicating better tenderness. FTIR analysis revealed higher α-helix content (37.54% vs. 33.50%) in Bian chicken, correlating with improved texture properties, while Cobb broiler showed higher lipid content (18.39% vs. 15.36%). GC×GC-TOF MS identified 2,150 flavor compounds in Bian chicken with 1,068 breed-specific compounds. The higher aldehyde content in Bian chicken (15.13% vs. 10.52%) contributed to its distinctive meaty aroma. Key discriminating compounds including (E)-2-nonenal, 2-pentyl-furan, heptanal, and 1-octen-3-ol were identified as potential biomarkers. These results align with broader consumer preferences in East Asia: Korean consumers perceived native chicken breeds as offering a more flavorful taste, juicier texture, and desirable chewiness, with breed information serving as a critical sensory cue influencing purchasing decisions. These discriminating compounds and FTIR-derived protein secondary-structure signatures can be used as phenotypic markers to support selection for flavor and tenderness in breeding programs. The combined application of FTIR and GC×GC-TOF MS technologies provides a comprehensive framework for poultry quality evaluation and supports the conservation and commercialization of indigenous chicken breeds like Bian chicken.

## Data Availability

The raw data supporting the conclusions of this article will be made available by the authors, without undue reservation.
